# Diagnostic Accuracy of Stool Tests for Colorectal Cancer Surveillance in Hodgkin Lymphoma Survivors

**DOI:** 10.3390/jcm9010190

**Published:** 2020-01-10

**Authors:** Berbel Ykema, Lisanne Rigter, Manon Spaander, Leon Moons, Tanya Bisseling, Berthe Aleman, Jan Paul de Boer, Pieternella Lugtenburg, Cecile Janus, Eefke Petersen, Judith Roesink, John Raemaekers, Richard van der Maazen, Iris Lansdorp-Vogelaar, Andrea Gini, Wieke Verbeek, Margriet Lemmens, Gerrit Meijer, Flora van Leeuwen, Petur Snaebjornsson, Beatriz Carvalho, Monique van Leerdam

**Affiliations:** 1 Department of Gastroenterology, Netherlands Cancer Institute, 1066 CX Amsterdam, The Netherlands; b.ykema@nki.nl (B.Y.); l.rigter@nki.nl (L.R.); w.verbeek@nki.nl (W.V.); 2 Department of Gastroenterology and Hepatology, Erasmus MC, University Medical Center, 3015 GD Rotterdam, The Netherlands; v.spaander@erasmusmc.nl; 3 Department of Gastroenterology and Hepatology, University Medical Center Utrecht, 3584 CX Utrecht, The Netherlands; l.m.g.moons@umcutrecht.nl; 4 Department of Gastroenterology and Hepatology, Radboud University Medical Center, 6525 GA Nijmegen, The Netherlands; tanya.bisseling@radboudumc.nl; 5 Department of Radiation Oncology, Netherlands Cancer Institute, 1066 CX Amsterdam, The Netherlands; b.aleman@nki.nl; 6 Department of Medical Oncology, Netherlands Cancer Institute, 1066 CX Amsterdam, The Netherlands; j.d.boer@nki.nl; 7 Department of Hematology, Erasmus MC Cancer Institute, 3015 GD Rotterdam, The Netherlands; p.lugtenburg@erasmusmc.nl; 8 Department of Radiation Oncology, Erasmus MC Cancer Institute, 3015 GD Rotterdam, The Netherlands; c.janus@erasmusmc.nl; 9 Department of Hematology, University Medical Center Utrecht, 3584 CX Utrecht, The Netherlands; e.j.petersen@umcutrecht.nl; 10 Department of Radiation Oncology, University Medical Center Utrecht, 3584 CX Utrecht, The Netherlands; j.m.roesink@umcutrecht.nl; 11 Department of Hematology, Radboud University Medical Center, 6525 GA Nijmegen, The Netherlands; johnmienraemaekers@gmail.com; 12 Department of Radiation Oncology, Radboud University Medical Center, 6525 GA Nijmegen, The Netherlands; richard.vandermaazen@radboudumc.nl; 13 Department of Public Health, Erasmus MC, 3015 GD Rotterdam, The Netherlands; i.vogelaar@erasmusmc.nl (I.L.-V.); a.gini@erasmusmc.nl (A.G.); 14 Department of Pathology, Netherlands Cancer Institute, 1066 CX Amsterdam, The Netherlands; m.lemmens@nki.nl (M.L.); g.meijer@nki.nl (G.M.); p.snaebjornsson@nki.nl (P.S.); b.carvalho@nki.nl (B.C.); 15 Department of Epidemiology, Netherlands Cancer Institute, 1066 CX Amsterdam, The Netherlands; f.v.leeuwen@nki.nl; 16 Department of Gastroenterology and Hepatology, Leiden University Medical Center, 2333 ZA Leiden, The Netherlands

**Keywords:** early detection of cancer, sensitivity and specificity, cancer survivors, Hodgkin lymphoma, colorectal cancer, multi-target stool test, fecal immunochemical testing (FIT)

## Abstract

Background: Hodgkin lymphoma (HL) survivors have an increased colorectal cancer (CRC) risk. Diagnostic accuracy of quantitative fecal immunochemical testing (FIT, OC Sensor) and/or a multi-target stool DNA test (mt-sDNA, Cologuard^®^) for advanced neoplasia (AN) was evaluated. Methods: 101 HL survivors underwent a surveillance colonoscopy and were asked to perform two stool tests (FIT and mt-sDNA). Advanced adenoma (AA), advanced serrated lesion (ASL), and AN (AA, ASL, CRC) were evaluated. Sensitivity, specificity, and area under the curve (AUC) for AN were calculated for different FIT cut-offs and mt-sDNA with colonoscopy as reference. Results: FIT and mt-sDNA were analyzed in 73 (72%) and 82 (81%) participants, respectively. AN was detected in 19 (26%) and 22 (27%), respectively. AN sensitivities for FIT cut-off of 10 ug Hb/g feces (FIT10) and mt-sDNA were 37% (95% confidence interval (CI): 16–62) and 68% (95% CI: 45–86), with corresponding specificities of 91% (95% CI: 80–97) and 70% (95% CI: 57–86), respectively. AUC for FIT was 0.68 (95% CI: 0.54–0.82) and for mt-sDNA 0.76 (95% CI: 0.63–0.89). Conclusions: In HL survivors, mt-sDNA showed highest sensitivity but with relatively low specificity for AN. Cost-effectiveness analyses is necessary to determine the optimal surveillance strategy.

## 1. Introduction

Hodgkin lymphoma (HL) survivors treated with abdominal radiotherapy and/or procarbazine-containing chemotherapy have an increased risk of developing colorectal cancer (CRC) with a relative risk reported between 2 and 7 [1,2,3,4,5,6]. A recent prospective colonoscopy study showed a high yield of advanced adenoma (AA) and advanced serrated lesions (ASL) in HL survivors at a younger age compared with the general population. Therefore, colonoscopy surveillance is recommended from the age of 35 or eight years after HL treatment [7,8]. Yet, colonoscopy is burdensome and has a small risk of serious complications [9,10,11]. For this reason, the accuracy of non-invasive surveillance modalities for HL survivors needs to be assessed. 

The fecal immunochemical test (FIT) is used for population-based CRC screening in the Netherlands [12,13,14,15,16]. This quantitative FIT detects human hemoglobin in feces and the positivity cut-off can be defined based on test performances. The FIT is easy to use and acceptance among the Dutch general population is high [17,18,19,20]. Participants with a positive stool test are referred for colonoscopy. An alternative stool test is the multi-target stool DNA test (mt-sDNA test, Exact Sciences, Madison, WI, USA). The latter detects, besides the presence of hemoglobin, altered DNA of colorectal neoplasia by sensitive analyses that target specific genetic and epigenetic biomarkers. Mt-sDNA has a higher sensitivity for detecting advanced colorectal neoplasia (AN—defined as AA, ASL, or CRC) compared to FIT [21,22,23,24,25]. FIT is currently considered inadequate for high-risk populations such as Lynch syndrome and individuals with familial risk of CRC, because of a relatively low sensitivity for AN. Therefore, these patients are offered periodic colonoscopy surveillance [26,27,28]. For HL survivors with a known increased risk of developing CRC, the effectiveness of stool tests has not yet been assessed. 

HL survivors might benefit from a more personalized approach for CRC surveillance due to the young age of developing AN, more right-sided lesions, other histology (high frequency of serrated polyps), and psychological burden of colonoscopy [7]. Stool tests could be an alternative for colonoscopy surveillance and could increase the participation rate of CRC surveillance in HL survivors. A higher participation rate will eventually result in a higher detection rate of AN, leading to a reduction of CRC incidence. 

This study will evaluate the diagnostic accuracy of FIT and/or mt-sDNA in HL survivors with colonoscopy as a reference. Stool test performance is one aspect to define the most optimal surveillance program. Based on stool test performance, further analyses can be performed to determine the optimal surveillance program for HL survivors being primary colonoscopy or stool test with a subsequent colonoscopy for participants with a positive test.

## 2. Materials and Methods

### 2.1. Study Population

Individuals were included in a prospective multicenter cohort study evaluating yield of colonoscopy in HL survivors. The study design and the diagnostic yield of colonoscopy in the study population were described previously [7,29]. Inclusion criteria for colonoscopy were infradiaphragmatic radiotherapy consisting of at least para-aortic and iliac fields, chemotherapy containing a cumulative procarbazine dosage of ≥2.8 g/m^2^ or infradiaphragmatic radiotherapy (any field(s) and chemotherapy (any regimen)). A total of 101 HL survivors underwent a colonoscopy between February 2015 and February 2017 at four Dutch study centers (the Netherlands Cancer Institute in Amsterdam, Erasmus MC Cancer Institute in Rotterdam, the University Medical Center in Utrecht, and Radboud University Medical Center in Nijmegen). Written informed consent was obtained from all participants who agreed to participate in the evaluation of the stool tests. Participants with an incomplete colonoscopy were excluded from analysis. The study was approved by the Medical Ethical Committee of the Netherlands Cancer Institute and was registered at the Dutch Trial Registry (ID NTR4961).

### 2.2. Stool Collection

Participants were invited to collect stool at home from one bowel movement prior to the start of the bowel preparation for colonoscopy. The two FIT tests as well as the mt-sDNA were performed on the same stool specimen with the request to deliver the stool within 60 h after collection (to minimalize the risk of DNA degradation). No restrictions were given for diet or medication prior to the collection.

### 2.3. Fecal Immunochemical Test

All the individuals included in the colonoscopy study were invited to perform two separate FIT tests (OC-Sensor, Eiken Chemical, Tokyo, Japan) on the same stool sample prior to colonoscopy. Participants received specific instructions on how to perform the FIT. The tip of the device should be stacked into the stool on three different spots and then put into the buffer in the test-kit. At the time of collection, participants were asked to annotate the date and time of collection. 

After stool sample collection, the two tests were sealed in plastic bags and stored in the fridge until the appointment for the colonoscopy. Once present at the colonoscopy center, the samples were sent to the laboratory, at the Netherlands Cancer Institute in Amsterdam, working based on the demands of ISO 15189 but not (yet) accredited. Upon arrival at the laboratory, FIT samples were stored at 20 °C and analyzed between 4 weeks after storage to avoid degradation of hemoglobin. Analysis was performed according to the manufacturer’s instruction by a trained technician, yielding quantitative measures of Hb concentration. 

As FIT is a quantitative assay, different pre-determined cut-offs for positivity could be evaluated, specifically 10, 15, and 20 µg Hb/g feces, referred to as FIT10, FIT15, and FIT20, respectively. Two FIT tests were evaluated and the highest measured concentration was used. 

### 2.4. Multi-Target Stool DNA Test

The multi-target stool DNA test (mt-sDNA, Cologuard^®^, Exact Sciences Corporation, Madison, WI, USA) was also analyzed. All colonoscopy participants received a container for stool sample collection and written information. Stool was collected in the provided container. The stool stabilization buffer (Exact Sciences, Madison, WI, USA) was added to the stool sample by the participant directly after sample collection (and after the two FIT tests were performed). On the lid of the container, the date and time of bowel movement was stated by the participant and the container was kept at room temperature until brought to the laboratory. 

The samples were processed in the laboratory with a final stool: buffer *w*/*v* ratio of 1:4, separated into aliquots, and stored at −80 °C until analysis. Exact Sciences Laboratory (Madison, WI, USA) performed the analyses of the mt-sDNA, as described before [24]. This laboratory is Clinical Laboratory Improvement Amendents (CLIA) licensed and College of American Pathologists (CAP) accredited. Mt-sDNA comprises, next to an immunochemical assay for human Hb, molecular assays for mutations of KRAS (in codon 12 referred to as KRAS1; and in codon 13 referred to as KRAS2) and epigenetic silencing (hypermethylation) of NDRG4 and BMP3 promoter regions. As a reference gene for human DNA quantity, β-actin is also included in this molecular assay. Of each marker, quantitative measurements were retrieved separately. For Hb, the cut-off used was 60 µg Hb/g feces (i.e., 600 ng Hb/mL buffer). A prespecified logistic regression algorithm combining the results of the different markers gives the final mt-sDNA result [24]. Calling of an individual test as positive or negative was done using the screening-validated threshold of 183 [24]. 

### 2.5. FIT and Mt-sDNA

The combination of both FIT10 (cut-off with highest sensitivity for FIT in detecting AN in our study population) and mt-sDNA tests were considered positive if at least one test was positive. For both tests analyses, the laboratory technicians were blinded for the colonoscopy results limiting investigator bias. 

### 2.6. Colonoscopy

The details of colonoscopy were described previously [29]. Colonoscopies were performed by experienced endoscopists. All detected polyps were described according to location, size, morphology, and macroscopic aspect and were directly removed for histological assessment according to standard protocol. The location of a polyp was classified as proximal when proximal to the splenic flexure. We analyzed the diagnostic accuracy of both stool tests according to the type of lesions detected during colonoscopy, for four groups: (1) any polyp (adenomas, serrated polyps), (2) AA (high-grade dysplasia, ≥25% villous component or ≥10 mm diameter), (3) ASL (dysplasia or ≥10 mm diameter), and (4) AN (AA, ASL, or CRC). All lesions were evaluated by the expert gastro-intestinal pathologist from the participating centers and all advanced lesions were reassessed by one expert pathologist (PS).

### 2.7. Outcome Measures and Statistical Analysis

A participant was considered to have a positive screen if at least one of the stool tests was considered positive at a specified cut-off level for both FIT and/or mt-sDNA tests at a single threshold. The reference was detection of colorectal lesions at colonoscopy. Furthermore, the sensitivity, specificity, positive predictive value (PPV), negative predictive value (NPV), positive likelihood ratio and negative likelihood ratio, and area under the curve (AUC) of the stool tests were evaluated for the four groups of lesions mentioned above (1–4). A 95% confidence interval (CI) was calculated for these parameters assuming binominal distribution. FIT10 and mt-sDNA were compared using the McNemar test. For the calculations of the performance of the stool tests with respect to polyp location, we used the most advanced lesion. This was classified in order of most advanced as 1. AA, 2. ASL, 3. non-advanced adenoma, and 4. non-advanced serrated lesion. If multiple advanced lesions were present (2 or more AA or ASL), then the largest was used as the most advanced lesion. Overall performance of FIT and mt-sDNA was evaluated by estimating the corresponding area under the receiver operating characteristic (ROC) curve. The ROC curve was represented by plotting the sensitivity versus 1 minus specificity. Values were compared using Chi square statistics. For analyses, SPSS V 22.0 was used. Results were reported according to the standards for reporting diagnostic accuracy [30].

## 3. Results

### 3.1. Study Population

**Figure 1:**
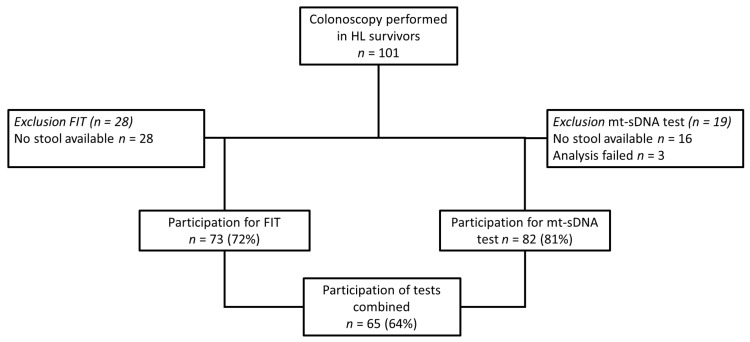
Study flow of Hodgkin lymphoma (HL) survivors who underwent a colonoscopy and participated in the fecal immunochemical test (FIT) and/or multi-target stool DNA test (mt-sDNA).

Between February 2015 and February 2017, 101 HL survivors underwent a colonoscopy [7]. Figure 1 shows the flow of participants eligible for this study. The median age at colonoscopy of participants was 51 years (range 32–73), and over half of participants were male. No difference in treatment category, time between HL treatment and colonoscopy were observed between FIT and/or mt-sDNA groups. An overview of the baseline characteristics of the study population of the FIT and/or mt-sDNA cohorts is shown in Table 1. 

### 3.2. Colonoscopy Results

The cecum was reached during colonoscopy in all FIT and mt-sDNA participants. The median inspection time during withdrawal was 20 min (range 8–70), Boston Bowel Preparation score > 6 was reported in all participants [7].

Table 1 shows the colonoscopy findings of FIT and/or mt-sDNA cohorts. In about one third of colonoscopy participants, no polyps were detected. AN was found in 26% (19 out of 73) of participants with an assessable FIT sample, 27% (22 out of 82) with an assessable mt-sDNA, and in 28% (18 out of 65) of participants with an assessable FIT and mt-sDNA. No CRC was found. One participant had both AA and ASL. Supplementary Table S1 shows the detection of colorectal neoplasia during colonoscopy in relation to FIT10 and/or mt-sDNA results. 

### 3.3. Fecal Immunochemical Test Results

Of 73 participants, 12 (16%) had a positive FIT result at a cut-off level of 10 ng/mL (FIT10). In the 12 FIT10 positive participants, any polyp was detected in 9 (75%), AA in 4 (33%), ASL in 3 (25%), and AN in 7 (58%). Among the 61 FIT10 participants with a negative test result, 12 (20%) presented with AN at colonoscopy. 

Table 2 summarizes the accuracy of FIT for any polyp, AA, ASL, and AN for three different cut-offs (FIT10, FIT15, and FIT20). FIT10 resulted in the highest sensitivity for AN of 37% (95% CI: 16–62) with a specificity of 91% (95% CI: 80–97). 

The PPV of FIT10 for detecting AN was 58% (95% CI: 34–80). Corresponding NPV was 80% (95% CI: 74-85). The area under the ROC curve of FIT for detecting AN was 0.68 (95% CI: 0.54–0.82). 

### 3.4. Mt-sDNA Results

Of the 82 participants with an evaluable mt-sDNA, 33 (40%) had a positive test. In these mt-sDNA positives, 28 (85%) participants had any polyp, 7 (21%) an AA, 9 (27%) an ASL, and 15 (46%) an AN (one participant had both AA and ASL). In the 49 mt-sDNA negatives, colonoscopy detected in 7 (14%) of the cases AN. 

The mt-sDNA sensitivity for AN was 68% (95% CI: 45–86) with a specificity of 70% (95% CI: 57–81). For ASL, mt-sDNA had an especially high sensitivity of 90% (95% CI: 56–98) with a corresponding specificity of 67% (95% CI: 55–77). The PPV and NPV for AN was 46% (95% CI: 34–57) and 86% (95% CI: 76–92), respectively. The positive and negative likelihood ratio for AN was 2.27 (95% CI: 1.41–3.67) and 0.45 (95% CI: 0.24–0.86), respectively. An overview is presented in Table 3. 

The AUC for ROC curve for detecting AN was 0.76 (95% CI: 0.63–0.89). The sensitivity of mt-sDNA is significantly higher compared to the sensitivity of FIT10 (*p* value 0.043).

### 3.5. FIT10 and/or Mt-sDNA Results Combined

The combination of a positive FIT10 and/or mt-sDNA resulted in 29 (45% of 65) positive results, of which 12 (41% of 29) had an AN. Of the 29 positive results, 22 (76%) had polyps, 6 (21%) had AA, 7 (24%) had ASL, and 12 (41%) had AN. Of the 36 participants with both test results negative, 6 (17%) had an AN. 

The sensitivity and specificity for detecting AN for combined tests were 67% (95% CI: 41–87) and 64% (95% CI: 49–77), respectively (Table 4). 

### 3.6. Diagnostic Accuracy of FIT and/or Mt-sDNA for the Most Advanced Lesion Per Participant Based on Location

FIT10 revealed a low sensitivity for the most advanced lesion, in the proximal colon location of only 13% (95% CI: 4–31) and in distal location of 23% (95% CI: 8–45). The highest sensitivity for proximal lesions was obtained with the mt-sDNA (56% (95% CI: 38–74)). For distal neoplasia, mt-sDNA had a sensitivity of 42% (95% CI: 22–63). The sensitivity of the FIT10 and/or mt-sDNA was 44% (95% CI: 22–69) for proximal location (Table 5).

## 4. Discussion

In this study, we evaluated the diagnostic accuracy for FIT and/or mt-sDNA in HL survivors treated with abdominal radiotherapy and/or procarbazine, with colonoscopy as a reference, in order to evaluate their potential as non-invasive surveillance modalities for HL survivors. We show that the mt-sDNA had the highest sensitivity for detecting AN in HL survivors (68%) with a corresponding specificity of 70% and FIT10 combined with mt-sDNA resulted in a sensitivity of 67% with a specificity of 64%. The sensitivity of FIT10 for detecting AN was only 37%, but specificity was higher (91%). Since this is a unique patient population, our findings are not generalizable to other groups.

It can be argued that the sensitivity of stool tests is more important than specificity in HL survivors with an indication for surveillance because of the high risk for CRC. Relative risks for CRC between 2 and 7 have been reported compared to the general population [1,2,3,4,31]. Aside from the increased risk for CRC, HL survivors also have a higher prevalence of (advanced) serrated lesions, serrated polyposis syndrome, and AA at a younger age compared to the general population [7]. Furthermore, colorectal lesions are more frequently proximal [7]. We evaluated whether stool tests can be used as a surveillance strategy with colonoscopy for stool test positive individuals. However, the optimal surveillance strategy and surveillance interval is not only defined by diagnostic test performance. A cost-effectiveness analysis can expand the knowledge about the optimal surveillance strategy, taking also participation into consideration.

Due to the increased risk of CRC, other criteria for optimal performance of stool tests should be used compared to an average-risk population-based screening [31,32,33,34,35,36]. In this high-risk-group, it can be argued that detection of precursor colorectal neoplasia is extra important due to the higher prevalence of these lesions and CRC. Little is known about the diagnostic accuracy of stool tests in high-risk groups.

Cost-effectiveness analysis showed that FIT10 (10 µg Hb/g feces) would be the most optimal cut-off level in the average risk population, with a high sensitivity for CRC [33]. The FIT10 sensitivity for detecting AN was low in HL survivors, around 40%, but comparable with sensitivity data in average risk and above-average risk populations [27,34,35,36]. 

We detected a higher sensitivity of 68% for AN using mt-sDNA compared to FIT10 in HL survivors. Presently, the mt-sDNA is being evaluated for surveillance in a high-risk population with previous CRC or adenomas/serrated polyps or with familial risk [37]. Previously, triennial mt-sDNA was considered not cost-effective in a cohort of previously unscreened 65-year-olds [38]. Of interest, in our study, a high sensitivity of 90% for mt-sDNA was found for ASL, which is higher than the 42% and 55% reported in prior publications [24,39]. Because of the high prevalence of ASL as detected during colonoscopy in HL survivors [7], this strengthens the usefulness of mt-sDNA in this patient group.

The mt-sDNA was off label use in HL survivors, but is interesting since the long-term effect of chemotherapy and/or radiation for these patients on the colonic epithelium, especially with respect to background methylation and cell turnover, has not been elucidated. 

Combining FIT10 and the mt-sDNA test resulted in similar sensitivity and specificity compared to the mt-sDNA test only. Therefore, this combination does not add to the test performance.

The mt-sDNA was more sensitive than FIT for detecting the most advanced lesions proximal in the colon in comparison to distal, while previously, a higher sensitivity for distal advanced neoplasia has been described [24]. This might be explained by the fact that more serrated lesions were found in this population and serrated lesions were mostly proximal located. For the most advanced lesions, FIT10 had a higher sensitivity for distal compared to proximal, which has also been reported in another study [24]. 

The main question is whether stool tests should be considered accurate enough to detect AN in this known high-risk population. Perhaps a higher diagnostic accuracy is needed in this group for detecting AN. When only performing a FIT10 in HL survivors, comparable to the current population-based CRC screening program in the Netherlands, 20% of AN would have been missed due to a false negative result. AN would still have been missed in 14% due to a negative result of mt-sDNA. Of note, this study only shows the results of one-time FIT and/or mt-sDNA. It has been proposed that repeating FIT and/or the mt-sDNA test (for instance every biennial or triennial testing) would result in a higher program sensitivity [40]. Further research is necessary to determine the most optimal surveillance interval.

Several advantages exist of performing stool tests as a first step in CRC surveillance in HL survivors. Firstly, stool tests could help to select which HL survivors should undergo a colonoscopy, especially in HL survivors unwilling to undergo a colonoscopy. Especially since it is known that cancer survivors generally undergo more medical interventions compared to the general population, including preventive measurements [41]. Additionally, undergoing a colonoscopy can be burdensome for the participant [10]. In our study, only 41% of eligible HL survivors agreed to undergo colonoscopy [7]. Providing a stool test could be an acceptable surveillance technique in HL survivors who do not want to undergo colonoscopy surveillance, thereby improving the participation rate in this population and eventually resulting in an increase of the AN detection rate. Of note, in our study population, the participation rate of performing stool test was acceptable (72% for FIT and 81% for mt-sDNA). We do not know what the participation of stool tests will be in the whole HL survivor group. We only have data from the colonoscopy participants. 

Directly performing a colonoscopy has been shown to reduce CRC incidence and mortality in other high-risk populations [42,43]. Precursor lesions of CRC can be endoscopically removed, thereby reducing the CRC incidence [14]. This strengthens the fact that colonoscopy is the first choice for colonoscopy surveillance in high-risk-groups, emphasizing the need in HL survivors, but currently participation is too low. 

This study has several strengths. To our knowledge, this is the first study to investigate the diagnostic accuracy of stool tests in cancer survivors with a known increased risk of developing CRC [1,2,5,7]. FIT has been evaluated in intermediate-risk individuals following colonoscopy screening and in individuals with a personal or familial history of CRC, but not in other established high-risk groups [27,44]. Another strength is that this population was screening-naïve and received both stool tests and a primary colonoscopy [29]. Previous screening can result in a lower prevalence of AN in a population and therefore impact test performance and especially yield lower positive predictive values [45]. Furthermore, researchers were blinded from results of the colonoscopy, which prevented investigator bias. 

This study has several limitations. Firstly, the sample size is small. In accordance with previous studies, the participation rate of HL survivors who underwent a colonoscopy was low [7,17,40]. Also, the colonoscopy itself as a reference is not 100% sensitive, since colorectal neoplasia can be missed [46]. 

Colonoscopy is considered a good surveillance modality for high-risk populations. However, stool tests may be a possible alternative surveillance modality for selection for colonoscopy in participants unwilling to undergo a colonoscopy. The main advantages are the non-invasive character of the test and a potential increased participation rate with a stool test compared to primary colonoscopy surveillance. A cost-effectiveness analysis is necessary to determine the optimal surveillance test for HL survivors being FIT, mt-sDNA test, or primary colonoscopy [38,47,48].

## 5. Conclusions

Sensitivity of FIT10 is limited for AN in HL survivors. The mt-sDNA test has a relative high sensitivity in detecting advanced neoplasia, especially for advanced serrated polyps. Stool tests can be used to select for colonoscopy as an alternative CRC surveillance modality in HL survivors, potentially increasing the participation rate in HL survivors unable or unwilling to undergo a colonoscopy. Cost-effectiveness analyses will be performed to determine the optimal strategy.

## Figures and Tables

**Table 1:** Patient characteristics of Hodgkin Lymphoma (HL) survivors who underwent a colonoscopy and performed a fecal immunochemical test (FIT) and/or multi-target stool DNA test (mt-sDNA).

Characteristic	FIT Participants (*n* = 73)	Mt-sDNA Participants (*n* = 82)	FIT and Mt-sDNA Participants (*n* = 65)
**Male gender, *n* (%)**	44 (60%)	45 (55%)	40 (62%)
**HL treatment category, %**			
Abdominal RT + procarbazine Procarbazine Abdominal RT	25 (34%) 35 (48%) 13 (18%)	29 (36%) 38 (46%) 15 (18%)	24 (37%) 28 (43%) 13 (20%)
**Time between HL treatment and colonoscopy, median (range), y**	23 (12–40)	23 (12–40)	23 (12–40)
**Age at colonoscopy, median (range), y**	51 (32–73)	51 (32–73)	50 (32–73)
**Neoplastic lesions in cohorts**			
No. per patient, median (range)	2 (0–21)	2 (0–28)	2 (0–21)
No. per patient, %			
0 ≥1	21 (29%) 52 (71%)	26 (32%) 56 (68%)	21 (32%) 44 (68%)
**Neoplasia detection per patient, %**			
Adenomas			
≥1 adenoma ≥1 advanced adenoma	43 (59%) 13 (18%)	44 (54%) 13 (16%)	36 (55%) 12 (19%)
Serrated polyps			
≥1 serrated polyps ≥1 advanced serrated lesion Advanced neoplasia	32 (44%) 7 (10%) 19 (26%)	35 (43%) 10 (12%) 22 (27%)	26 (40%) 7 (11%) 18 (28%)

**Table 2:** Performance of fecal immunochemical test (FIT) at different cut-off levels for different disease outcomes.

	Number of Events/Total	Sensitivity	Specificity	PPV	NPV	LR+	LR−
		(%, 95% CI)	(%, 95% CI)	(%, 95% CI)	(%, 95% CI)	(95% CI)	(95% CI)
**FIT10**							
Any polyp	52/73	17 (8–30)	86 (64–97)	75 (47–91)	30 (25–34)	1.21 (0.36–4.04)	0.96 (0.78–1.20)
AA	13/73	31 (9–61)	87 (75–94)	33 (15–59)	85 (80–89)	2.31 (0.82–6.53)	0.80 (0.55–1.16)
ASL	7/73	43 (10–82)	86 (76–94)	25 (10–49)	93 (88–96)	3.14 (1.10–8.97)	0.66 (0.35–1.27)
AN	19/73	37 (16–62)	91 (80–97)	58 (34–80)	80 (74–85)	3.98 (1.43–11.05)	0.70 (0.49–0.99)
**FIT15**							
Any polyp	52/73	14 (6–26)	86 (64–97)	70 (40–89)	29 (25–33)	0.94 (0.27–3.30)	1.01 (0.82–1.24)
AA	13/73	31 (9–61)	90 (70–96)	40 (18–67)	86 (81–90)	3.08 (1.01–9.37)	0.77 (0.53–1.12)
ASL	7/73	29 (4–71)	88 (78–95)	20 (6–49)	92 (88–95)	2.36 (0.62–9.00)	0.81 (0.50–1.31)
AN	19/73	32 (13–57)	93 (82–98)	60 (32–83)	79 (74–84)	4.26 (1.35–13.49)	0.74 (0.54–1.01)
**FIT20**							
Any polyp	52/73	12 (4–23)	91 (70–99)	75 (40–93)	29 (26–33)	1.21 (0.27–5.53)	0.98 (0.82–1.16)
AA	13/73	23 (5–54)	92 (82–97)	38 (14–69)	85 (81–88)	2.77 (0.75–10.16)	0.84 (0.62–1.14)
ASL	7/73	29 (4–71)	91 (81–97)	25 (8–57)	92 (88–95)	3.14 (0.78–12.72)	0.79 (0.49–1.26)
AN	19/73	26 (9–51)	94 (85–99)	63 (31–86)	79 (73–83)	4.74 (1.25–17.95)	0.78 (0.59–1.03)

Any polyp = non-advanced and advanced adenoma and serrated polyp; AA = advanced adenoma; ASL = advanced serrated lesion; AN = advanced neoplasia; CI = confidence interval; PPV = positive predictive value; NPV = negative predictive value; LR+ = likelihood ratio positive; LR− = likelihood ratio negative; FIT10 (≥10 µg Hb/g feces); FIT15 (≥15 µg Hb/g feces); FIT20 (≥20 µg Hb/g feces).

**Table 3:** Performance of multi-target stool DNA test (mt-sDNA).

	Number of Events/Total	Sensitivity	Specificity	PPV	NPV	LR+	LR−
		(%, 95% CI)	(%, 95% CI)	(%, 95% CI)	(%, 95% CI)	(95% CI)	(95% CI)
**Mt-sDNA**							
Any polyp	56/82	50 (36–67)	81 (61–93)	85 (71–93)	43 (35–51)	2.60 (1.13–5.96)	0.62 (0.45–0.85)
AA	13/82	54 (25–81)	62 (50–74)	21 (13–33)	88 (79–93)	1.43 (0.79–2.57)	0.74 (0.40–1.37)
ASL	10/82	90 (56–98)	67 (55–67)	27 (20–36)	98 (88–100)	2.70 (1.83–3.97)	0.15 (0.02–0.97)
AN	22/82	68 (45–86)	70 (57–81)	46 (34–57)	86 (76–92)	2.27 (1.41–3.67)	0.45 (0.24–0.86)

Any polyp = non-advanced and advanced adenoma and serrated polyp; AA = advanced adenoma; ASL = advanced serrated lesion; AN = advanced neoplasia; CI = confidence interval; PPV = positive predictive value; NPV = negative predictive value; LR+ = likelihood ratio positive; LR− = likelihood ratio negative.

**Table 4:** Performance of fecal immunochemical test at cut-off of ≥10 µg Hb/g feces (FIT10) and multi-target stool DNA test (mt-sDNA) combined.

	Number of Events/ Total	Sensitivity (%, 95% CI)	Specificity (%, 95% CI)	PPV (%, 95% CI)	NPV (%, 95% CI)	LR+ (95% CI)	LR− (95% CI)
**Combined stool test**							
Any polyp	24/65	50 (35–65)	66 (43–85)	76 (62–86)	39 (29–49)	1.50 (0.77–2.94)	0.75 (0.49–1.14)
AA	12/65	50 (21–79)	57 (42–70)	21 (12–33)	83 (73–90)	1.15 (0.61–2.19)	0.88 (0.48–1.63)
ASL	7/65	100 (59–100)	62 (48–75)	24 (19–31)	100 *	2.64 (1.90–3.66)	0
AN	18/65	67 (41–87)	64 (49–77)	41 (30–54)	83 (72–91)	1.84 (1.12–3.04)	0.52 (0.26–1.04)

Any polyp = non-advanced and advanced adenoma and serrated polyp; AA = advanced adenoma; ASL = advanced serrated lesion; AN = advanced neoplasia; CI = confidence interval; PPV = positive predictive value; NPV = negative predictive value; * = calculation not possible (due to no false negative results); LR+ = likelihood ratio positive; LR- = likelihood ratio negative; FIT10 (≥10 µg Hb/g feces).

**Table 5:** Accuracy of fecal immunochemical test at cut-off of ≥10 µg Hb/g feces (FIT10) and/or multi-target stool DNA test (mt-sDNA) according to location of most advanced neoplasia.

	Sensitivity (%, 95% CI)	Specificity (%, 95% CI)
Proximal neoplasia *		
FIT10	13 (4–31)	77 (55–92)
Mt-sDNA	56 (38–74)	58 (37–78)
FIT10 and mt-sDNA	54 (33–73)	56 (31–78)
Distal neoplasia *		
FIT10	23 (8–45)	87 (69–96)
Mt-sDNA	42 (22–63)	44 (26–62)
FIT10 and mt-sDNA	44 (22–69)	46 (27–67)

* Proximal neoplasia = proximal to (and including) flexura lienalis; Distal neoplasia = Distal to flexura lienalis; FIT10 (≥10 µg Hb/g feces).

## Supplementary Materials

The following are available online at https://www.mdpi.com/2077-0383/9/1/190/s1, Table S1: Yield of colonoscopy in relation to FIT10, FIT15, FIT20, mt-sDNA and FIT10 and/or mt-sDNA result combined.
