# Supplementary material for: Diagnostic Accuracy of Stool Tests for Colorectal Cancer Surveillance in Hodgkin Lymphoma Survivors
Source: J Clin Med. 2020 Jan 10;9(1):190. doi: 10.3390/jcm9010190 (PMC7019558; doi:10.3390/jcm9010190)
Supplement: Supplementary file 1 [file jcm-09-00190-s001.pdf]

SUPPLEMENTARY MATERIALS

Supplementary table 1. Yield of colonoscopy in relation to FIT10, FIT15, FIT20, mt-sDNA and FIT10 and/or mt-sDNA result combined.

	**FIT10+** (n, %)	**FIT10-** (n, %)	**FIT15+** (n, %)	**FIT15-** (n, %)	**FIT20+** (n, %)	**FIT20-** (n,%)	**Mt-sDNA +** (n, %)	**Mt-sDNA -** (n, %)	**FIT10 and/or mt-sDNA +** (n, %)	**FIT10 and/or mt-sDNA -** (n, %)
**Any polyp**										
Present	9 (17.3%)	43 (82.7%)	7 (13.5%)	45 (86.5%)	6 (11.5%)	46 (88.5%)	28 (50.0%)	28 (50.0%)	22 (50.0%)	22 (50.0%)
Not present	3 (14.3%)	18 (85.7%)	3 (14.3%)	18 (85.7%)	2 (9.5%)	19 (90.5%)	5 (19.2%)	21 (80.8%)	7 (33.3%)	14 (66.7%)
**AA**										
Present	4 (30.8%)	9 (69.2%)	4 (30.8%)	9 (69.2%)	3 (25.1%)	10 (76.9%)	7 (58.3%)	6 (46.2%)	6 (50.0%)	6 (50.0%)
Not Present	8 (13.3%)	52 (86.7%)	6 (10.0%)	54 (90.0%)	5 (8.3%)	55 (91.7%)	26 (37.7%)	43 (62.3%)	23 (43.4%)	30 (56.6%)
**ASL**										
Present	3 (42.9%)	4 (57.1%)	2 (28.6%)	5 (71.4%)	2 (28.6%)	5 (71.4%)	9 (90.0%)	1 (10.0%)	7 (100%)	0 (0%)
Not present	9 (13.6%)	57 (86.4%)	8 (12.1%)	58 (87.9%)	6 (9.1%)	60 (90.9%)	24 (33.3%)	48 (66.7%)	58 (37.9%)	36 (62.1%)
**AN**										
Present	7 (36.8%)	12 (63.2%)	6 (31.6%)	13 (68.4%)	5 (26.3%)	14 (73.7%)	15 (68.2%)	7 (31.8%)	12 (66.7%)	6 (33.6%)
Not present	5 (9.3%)	49 (90.7%)	4 (7.4%)	50 (92.6%)	3 (5.6%)	51 (94.4%)	18 (30.0%)	42 (70.0%)	17 (36.2%)	30 (63.8%)

Any polyp = non-advanced and advanced adenoma and serrated polyp; AA = advanced adenoma; ASL = advanced serrated polyp; AN = advanced neoplasia; FIT10 (≥10 μg Hb/g faeces); FIT15 (≥15 μg Hb/g faeces); FIT20 (≥20 μg Hb/g faeces).
